# Fungal pathogens and symbionts: Living off the fat of the land

**DOI:** 10.1371/journal.ppat.1012551

**Published:** 2024-09-26

**Authors:** Olga A. Nev, Márcia David-Palma, Joseph Heitman, Alistair J. P. Brown, Marco A. Coelho

**Affiliations:** 1 Medical Research Council Centre for Medical Mycology, University of Exeter, Exeter, United Kingdom; 2 Department of Molecular Genetics and Microbiology, Duke University Medical Center, Durham, North Carolina, United States of America; University of Michigan, UNITED STATES OF AMERICA

## Introduction

Here, we discuss the independent loss of de novo fatty acid biosynthetic capacity in evolutionarily divergent fungal species that have developed (or appear to be developing) obligate relationships with their hosts.

The fungal kingdom encompasses a vast diversity of species that significantly influence Earth’s ecosystems, both supporting and challenging their balance. Some fungi have developed additional metabolic pathways that expand the core metabolic network that links central carbon metabolism with amino acid, nucleotide, and lipid anabolism, thus having an enhanced fitness in specific niches. Other fungi have taken an opposite evolutionary route, becoming highly specialized and downsizing their core metabolic network [1,2].

At one end of this spectrum, species such as the white-rot fungi have developed a metabolic machinery that enables lignin degradation [3], and the human pathogen *Candida albicans* exhibits an expansion of gene families involved in nutrient acquisition (for instance, secreted proteases, lipases, and phospholipases) and nutrient uptake (for instance, transporters and amino acid permeases) [4], thus facilitating nutrient scavenging in host niches. Tight regulation of such “metabolic extensions” in an environmentally contingent manner provides these fungi with the metabolic flexibility to navigate complex fitness landscapes as they shift from one microenvironment to another. Notable examples include the control of lignin degradation in white-rot fungi [3], secreted hydrolases in *C*. *albicans* [4], and micronutrient assimilation in the basidiomycete *Cryptococcus neoformans*, and the ascomycetes *Aspergillus fumigatus* and *C*. *albicans* [5]. Despite being separated by hundreds of millions of years of evolution, *C*. *neoformans* and *A*. *fumigatus* are both capable of rapid metabolic adaptation, enabling efficient transitions between environmental and human niches [6,7].

**Fig 1:**
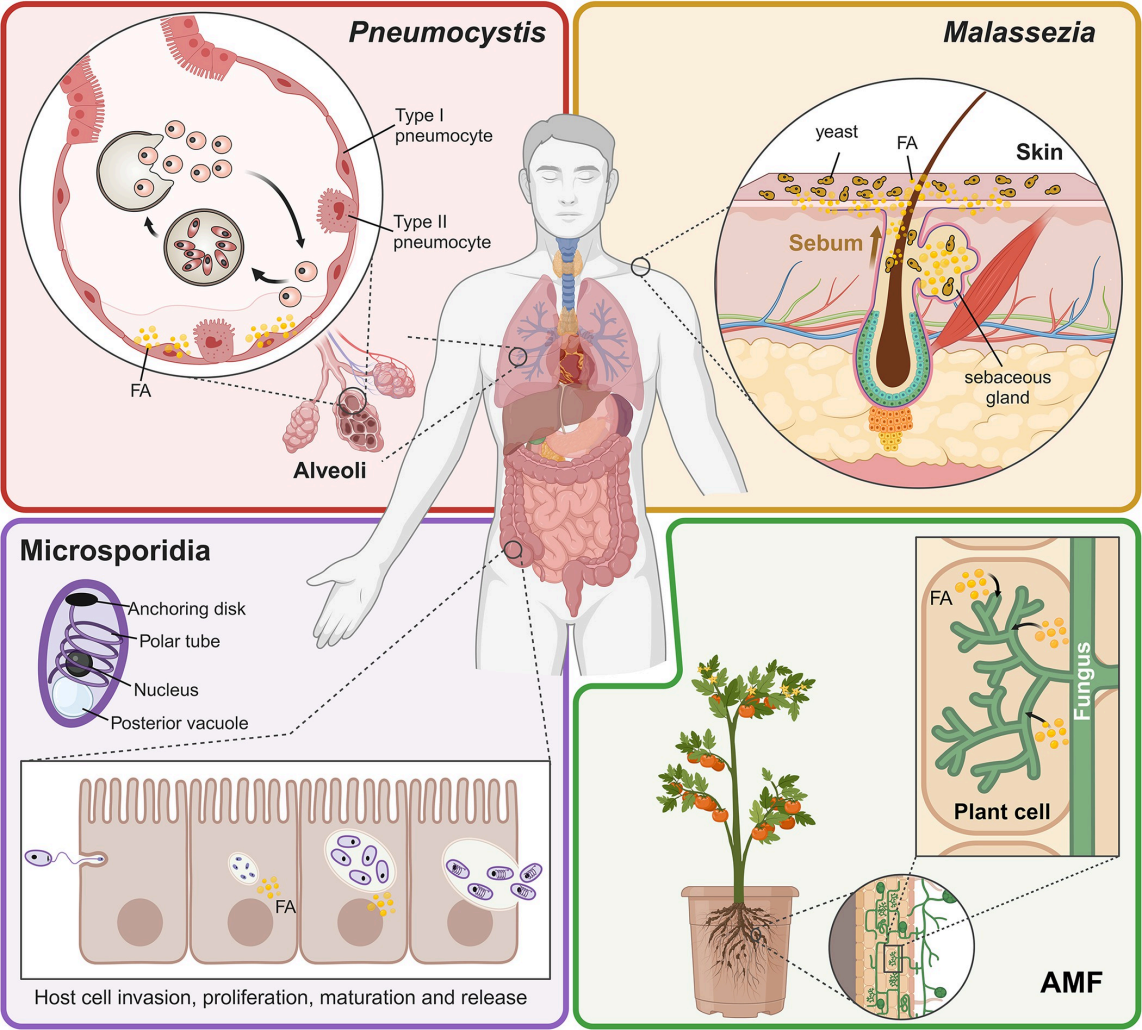
Distinct fungal–host adaptations resulting in loss of de novo fatty acid synthesis. This figure highlights diverse adaptations of fungi that rely on host-derived fatty acids (FAs) for growth due to the loss of de novo FA synthesis: *Pneumocystis* spp., residing in mammalian lungs becoming pathogenic when host immunity is compromised, are shown from haploid cell conjugation to zygote formation, mature cyst development with elongated trophic forms, and spore dispersion. *Malassezia* spp., living on skin, utilize FAs from sebum, depicted with yeast cells in sebaceous gland secretions, where overgrowth can lead to skin diseases. Microsporidia, as parasites in the gastrointestinal tract, progress through spore resistance in the environment, germination, host cell invasion via polar tubule eversion, sporoplasm injection, proliferation and maturation, and spore release to infect new cells. Arbuscular mycorrhizal fungi (AMF) engage in symbiotic relationships with plant roots, depicted with fungal structures inside plant cells where they receive lipids from the plant, compensating for their inability to synthesize FAs themselves. Figure generated with BioRender (https://www.biorender.com/).

At the other end of this spectrum, other fungal pathogens have taken the opposite evolutionary path, becoming highly specialized rather than promoting robustness through adaptability. For instance, *Pneumocystis*, *Malassezia*, and *Encephalitozoon* species have undergone significant genome reductions [8–11], shedding metabolic functions that became redundant as they grew increasingly dependent upon their host for nutrients. Despite being highly divergent, belonging to different fungal phyla (the Ascomycota, Basidiomycota, and Microsporidia, respectively), and colonizing different niches in humans (lung, skin, and gastrointestinal tract, respectively) (Fig 1), these species share a common evolutionary hallmark: the loss of genes essential for fatty acid synthesis [8,9,12]. This is also the case for the arbuscular mycorrhizal fungi (AMF) of the subphylum Glomeromycotina [13], as well as for many bacterial symbionts [14], suggesting a convergent evolutionary strategy.

## Fatty acid synthesis

Fatty acids are (1) integral components of cell membranes, (2) key energy stores, (3) precursors of signaling molecules, and (4) substrates for posttranslational modification of proteins. The fatty acid biosynthetic pathway is ubiquitous across all kingdoms of life and is essential for free-living fungi. It starts with the conversion of acetyl-coenzyme A (CoA) to malonyl-CoA by acetyl-CoA carboxylase (ACC). In *Saccharomyces cerevisiae*, the cytosolic ACC is encoded by *ACC1* [15]. The nascent fatty acid chain is then extended 2 carbons at a time using malonyl-CoA as the substrate, in a series of cyclic reactions catalyzed by fatty acid synthase (FAS). In *S*. *cerevisiae* and *Schizosaccharomyces pombe*, the *FAS1* and *FAS2* genes encode the trifunctional β and tetrafunctional α subunits, respectively, of the type I α_6_β_6_ FAS complex located in the cytoplasm [15]. Corresponding type II FAS activities are expressed in the mitochondrion. Cytosolic FAS synthesizes fatty acid chains up to 20 carbons long, with further extension up to 26 carbons occurring in the endoplasmic reticulum by specific elongases [15].

Fatty acid synthesis is energetically costly. In yeast, most fatty acids are 16 or 18 carbons long, and each cycle of 2-carbon extension consumes 1 ATP and 2 NADPH^+^ molecules [16]. Consequently, rather than synthesizing fatty acids, scavenging them from fatty acid–rich microenvironments offers an energetically attractive alternative that reduces the metabolic costs associated with cell growth and division. This cost–benefit scenario is underscored by the lack of retention of *FAS* genes in highly divergent fungal species that are adapting to, or have already established, obligate relationships with their hosts (Fig 1). This pattern does not necessarily reflect direct selection for gene loss but rather represents an adaptive outcome for thriving in environments rich in fatty acids. Essentially, these species have evolved to “live off the fat of the land,” leveraging externally sourced fatty acids to optimize their energy efficiency.

## Independent losses of fatty acid synthetic capacity

*Pneumocystis* species are prime examples of obligate pathogens that have lost the capacity to synthesize fatty acids. These pathogens have become highly specialized, with different *Pneumocystis* species infecting humans, Old World primates, New World primates, and other mammals [17]. *Pneumocystis jirovecii* infects humans, causing pneumocystis pneumonia (PCP) that results in over 400,000 deaths annually among immunocompromised patients. The dominant fatty acids in this fungus, which resides within the hypophase of alveoli in the lung [18], are palmitic (16 carbons: 0 double bonds), stearic (18:0), and oleic (18:1) acids [19]. Yet, *P*. *jirovecii* lacks both the *FAS1* and *FAS2* genes [8] and hence lacks the cytosolic fatty acid synthesis (FAS) pathway. The rodent-associated *Pneumocystis* species (*P*. *carinii* and *P*. *murina*) retain the mitochondrial FAS pathway, comprised of 8 bacterial-like enzymes, which are normally important for mitochondrial biogenesis and respiration [15,20]. However, *P*. *jirovecii* lacks 1 key mitochondrial FAS gene (*MCT1*), which strongly suggests that this pathogen must assimilate essential fatty acids from its human host, since the loss of key genes on a pathway (not the whole pathway) is sufficient to confer loss of de novo synthesis. Indeed, *Pneumocystis* has been shown to assimilate cholesterol and certain fatty acids (for instance, palmitic, stearic, and oleic fatty acids) from the medium to generate phospholipids and form new membranes [21,22]. Lung surfactant is composed mainly of lipids, which presumably promotes *Pneumocystis* lipid acquisition strategies.

*Malassezia* species, the predominant fungi on the skin of humans and other warm-blooded animals, thrive in lipid-rich sebaceous sites [23]. Unique among skin-colonizing fungi, they lack the cytosolic *FAS1* and *FAS2* genes essential for autonomously synthesizing long-chain fatty acids and instead rely on external sources such as sebum and epidermal lipids for growth [9] (Fig 1). Reflecting this adaptation, *Malassezia* genomes are among the smallest for free-living fungi (approximately 7 to 9 Mb) yet boast an expanded enzymatic repertoire for lipid exploitation, including various genes encoding secreted lipases, phospholipases, and sphingomyelinases [24–26]. These enzymes are crucial for breaking down abundant extracellular lipids in the skin into fatty acids, which *Malassezia* then utilizes for growth. However, the residual unsaturated fatty acids left behind may induce skin inflammation, linking *Malassezia* to skin diseases such as dandruff and eczema [27]. This enzymatic activity, characterized by varied enzyme copy numbers and repertoires among different *Malassezia* species, leads to distinctive patterns of lipid consumption and utilization, reflecting their adaptation to diverse skin environments and contributing to niche specificity [28]. Additionally, engineered *Malassezia furfur* strains undergoing hyphal development show increased lipase expression, potentially further enhancing skin colonization and the pathogenic potential of *Malassezia* [10].

The Microsporidia are unconventional microbial pathogens that infect mammals, fish, insects, and myriad other hosts. Originally thought to be parasites given their unusual properties, molecular phylogenetic studies have reclassified them as fungi, with origins linked to the early-diverged aquatic fungal lineages (the Cryptomycota/Rozellomycota) [11]. As obligate microbes, Microsporidia can only be grown in their hosts or cells derived from them, and not in axenic culture on a petri dish or in culture media. These pathogens have highly reduced genomes [12,29] and, similar to *Pneumocystis*, *Malassezia*, and AMF, have lost FAS and thus must scavenge lipids from their host (Fig 1). They also harbor an unusual mitochondrion, called the mitosome, which lacks the ability to provide ATP to its host cell. Instead, Microsporidia have acquired ATP transporters that enable them to scavenge ATP from their host cell cytoplasm [30]. While much remains to be learned about these fascinating microbes, this shared evolutionary facet (loss of FAS) with other fungi provides unique insights into their obligate intracellular lifestyle.

When plants first exited the oceans to colonize the planet as terrestrial organisms, a close symbiotic relationship was forged with fungal symbionts that enabled early land plants to efficiently acquire nutrients from the soil [31]. An example of such an interaction occurs between AMF and the roots of host plants. This symbiosis is highly reciprocal: AMF extract nitrogen and phosphate from the soil and transfer these nutrients to their plant hosts, which in return provide lipids to the fungal partner [32]. Consequently, AMF species that are among the most common fungal symbionts of plants have, like the human pathogens *Pneumocystis* and *Malassezia*, lost their FAS genes [33], making them reliant on their hosts for survival. Interestingly, AMF can also source lipids from bacterial symbionts, which then serve as an energy source via fatty acid beta-oxidation [34].

## Potential fatty acid scavenging mechanisms

How do these fungi assimilate fatty acids from their hosts? AMF appear to express AMP-binding domain proteins (RiFAT1 and RiFAT2, in *Rhizophagus irregularis*), which show sequence similarity to the *S*. *cerevisiae* fatty acid transporter Fat1. These transporters seem to take up myristic acid (C14:0) and palmitic acid (C16:0) that have been exported across the peri-arbuscular membrane by the host plant [35]. In *Malassezia*, in silico studies have identified several genes potentially involved in the import and activation of fatty acids that have been released by secreted lipases, phospholipases, and sphingomyelinases from lipids produced by host skin sebaceous glands [28]. These genes encode orthologs of *S*. *cerevisiae* fatty acyl-CoA synthetases (ACSs) Faa1-4 and Fat1-2, which are tailored to process fatty acids of different chain lengths [28]. Recent findings suggest that *Malassezia* Faa1 orthologs facilitate the uptake of C14-C16 fatty acids essential for growth [36]. Meanwhile, *Pneumocystis* species appear to have maintained orthologs of *S*. *cerevisiae* Acb1, Faa1, and Fat1, which are potentially involved in fatty acid activation and import [37], and have been shown experimentally to assimilate certain fatty acids from the host and incorporate them into complex phospholipids [21,38]. The endocytosis of membranous material represents another possible means by which *Pneumocystis* might assimilate fatty acids. Indeed, genomic analysis indicates the preservation of proteins related to clathrin-dependent endocytosis in *Pneumocystis* [37], and electron microscopy studies have revealed a tight association of *Pneumocystis* with host cells, consistent with the potential exploitation of endocytosis as a lipid uptake pathway [39].

## Implications and future directions

The genome reductions and specializations displayed by *Pneumocystis*, *Malassezia*, Microsporidia, and AMF pose significant technical challenges for their experimental analysis. While axenic culture methods for *Pneumocystis* have been reported, they have been hard to reproduce [18,40]. This has represented a major obstacle in *Pneumocystis* research, leading to the significant delays in sequencing *Pneumocystis* genomes, for example. Similarly, Microsporidia can only be cultured in vitro with the support of host cells [41], and only a limited number of AMF species been grown in axenic culture [42,43]. *Malassezia* spp., even with necessary lipid supplementation, are also challenging to cultivate. These yeasts are fastidious, slow-growing, and their isolation from polymicrobial samples is often hampered by the presence of other fast-growing microorganisms [44]. Among them, *Malassezia pachydermatis*, *Malassezia sympodialis*, and *M*. *furfur* are the least demanding, with *M*. *pachydermatis* being able to grow on Sabouraud-dextrose agar containing only trace amounts of lipids from peptone, indicating its efficiency in utilizing available lipids [26]. In contrast, *Malassezia globosa* and *Malassezia restricta* require specific lipid supplementation and extended incubation periods [45]. This difficulty in cultivating *Malassezia* underscores a significant gap in our understanding of its true diversity. Indeed, recent advances in direct sequencing have uncovered a broad ecological presence of *Malassezia* across diverse environments, from soil to deep-sea vents [46], suggesting their ecological roles and adaptive strategies may be vastly underappreciated. These findings invite further investigation into their origins, specific adaptations, and potential transitions between marine and terrestrial environments. The relevance of these insights is amplified by recent studies linking these yeasts with systemic disorders such as Crohn’s disease [47] and certain cancers [48] and their ability to inhibit other skin pathogens, such as *Candida auris* [49,50] and *Staphylococcus aureus* [51].

The development of robust axenic culture conditions for these medically and agriculturally important fungal species represents an important goal. Bioinformatic analyses of genome sequences have identified likely auxotrophic requirements [8,12,33], and further insights are expected from in-depth metabolic modeling. Robustly cultivating these fungal species independently of their hosts would further enhance our ability to dissect their molecular and genetic mechanisms and deepen our understanding of their dependencies and interactions with their hosts. From a clinical perspective, establishing axenic culture conditions would accelerate the development of better diagnostics, vaccines, and more effective antifungal therapies, all of which are essential for combating infections caused by *Pneumocystis*, *Malassezia*, and Microsporidia.
